# Association Between Visit-to-Visit Variability in Blood Pressure and Cardiovascular Events in Hypertensive Patients After Successful Percutaneous Coronary Intervention

**DOI:** 10.14740/jocmr2173w

**Published:** 2015-05-08

**Authors:** Kouki Gondo, Shin-ichiro Miura, Yasunori Suematsu, Yuhei Shiga, Takashi Kuwano, Makoto Sugihara, Amane Ike, Atsushi Iwata, Kota Motozato, Takaaki Kusumoto, Hiroaki Nishikawa, Keijiro Saku

**Affiliations:** a Department of Cardiology, Fukuoka University School of Medicine, Fukuoka, Japan; b Department of Molecular Cardiovascular Therapeutics, Fukuoka University School of Medicine, Fukuoka, Japan; c Division of Cardiology, Izumi General Medical Center, Kagoshima, Japan

**Keywords:** Visit-to-visit variability in blood pressure, Percutaneous coronary intervention, Major adverse cardiovascular events, Myocardial infarction, Acute coronary syndrome

## Abstract

**Background:**

Visit-to-visit variability (VVV) in blood pressure (BP) in addition to high BP has been shown to be a strong predictor of coronary events and stroke. Therefore, we investigated the associations between VVV in BP or BP levels and cardiovascular events after successful percutaneous coronary intervention (PCI).

**Methods:**

We enrolled 176 hypertensive patients who had undergone successful PCI and who had four clinic visits to measure BP until follow-up coronary angiography (CAG) at 6 - 9 months after PCI. The patients were divided into those with acute coronary syndrome (ACS group; n = 50) and those with stable angina pectoris (SAP group; n = 126). We determined VVV in BP expressed as the standard deviation (SD) of average BP, average, and the maximum and minimum BP during the follow-up period. Major adverse cardiovascular events (MACEs) (myocardial infarction (MI), target lesion revascularization (TLR) and all-cause death) were also analyzed.

**Results:**

There were no significant differences in VVV in BP, average BP or maximum or minimum BP between the patients with and without MACE in all patients, the ACS and SAP groups. Interestingly, in the ACS group, VVV in SBP and maximum SBP in patients with MI were significantly higher than those in patients without MI. The cut-off levels for VVV in BP and maximum SBP that gave the greatest sensitivity and specificity for MI in the ACS group were 15.1 and 138 mm Hg, respectively.

**Conclusion:**

Higher VVV in SBP and maximum SBP in patients with ACS after successful PCI were associated with the onset of MI.

## Introduction

High blood pressure (BP) is a major risk factor for the onset and progression of cardiovascular disease (CVD) and stroke. Recently, visit-to-visit variability (VVV) in BP has also been shown to be a strong predictor of CVD, stroke and mortality independent of BP [1-4], although there is some controversy [5, 6]. The most noteworthy report was a systematic review and meta-analysis published in Hypertension in 2014 [4]. Diaz et al stated that modest associations between VVV in BP and CVD and all-cause mortality were present in many published studies. An increase in VVV in BP may be explained by arterial stiffness and abnormal autonomic function [7, 8]. Furthermore, heart rate variability, a measure of autonomic dysfunction, has been associated with an increased risk of myocardial ischemia in CVD patients [9]. Thus, VVV in BP is probably associated with CV events due to severe arterial stiffness and abnormal autonomic function.

According to the Japanese Society of Hypertension Guidelines for the Management of Hypertension (JSH2014), the target in BP control should be < 140/90 mm Hg [10]. When patients have multiple coronary risk factors, JSH2014 recommends a lower BP level (< 130/80 mm Hg) in the absence of significant coronary artery stenosis [10]. Although this point is still controversial, a decrease in BP causes a reduction in the diastolic coronary perfusion pressure and induces myocardial ischemia, which worsens the prognosis (J-shaped curve phenomenon) [11-13]. Nonetheless, there is no recommendation regarding the optimal lowest level or VVV in BP in patients with CVD.

To prevent the occurrence and progression of CVD, it may be important to manage both VVV in BP in addition to optimal BP in hypertensive patients with successful percutaneous coronary intervention (PCI), and we hypothesized that VVV in BP in hypertensive patients associates with major adverse cardiovascular events (MACEs) after PCI. Therefore, we investigated the associations between VVV in BP or BP levels and MACE after successful PCI.

## Methods

### Study population

We enrolled 176 consecutive hypertensive patients who had undergone successful PCI and who had four clinic visits to measure BP until follow-up coronary angiography (CAG) at 6 - 9 months after PCI. The patients were divided into those with acute coronary syndrome (ACS group; n = 50) and those with stable angina pectoris (SAP group; n = 126). Our protocol was approved by the Ethics Committee of Fukuoka University Hospital (IRB #14-12-06). We retrospectively collected and analyzed all data using the database of Fukuoka University Hospital.

### CAG

CAG and PCI were performed according to the Judkins technique by the patients’ interventional cardiologists [14]. Coronary angiograms were analyzed with respect to the 15-segment coding system of the American Heart Association [15], and significant stenosis or restenosis was considered to be > 50% diameter stenosis. Patients underwent stent implantation (bare-metal or drug-eluting stent (BMS or DES)) and plain old balloon angioplasty (POBA) based on the judgment of their cardiologists.

### Assessment of clinical outcomes during the follow-up period

MACEs (myocardial infarction (MI), target lesion revascularization (TLR) and all-cause death) were analyzed throughout the follow-up period. For a diagnosis of MI, the patient had to have shown both evidence of ischemic electrocardiogram changes and elevation of cardiac enzymes. TLR was performed if the lesion had significant luminal stenosis (> 50% diameter stenosis) in the presence of angina symptoms and/or proven myocardial ischemia in the target vessel, or in follow-up CAG. All-cause death was identified during the follow-up period.

### Assessment of cardiovascular risk factors

In all subjects, we measured systolic BP (SBP), diastolic BP (DBP), and serum levels of triglycerides (TG), high-density lipoprotein cholesterol (HDL-C), low-density lipoprotein cholesterol (LDL-C), estimated glomerular filtration rate (eGFR), uric acid (UA), and hemoglobin A1c (HbA1c). All blood samples were drawn in the morning after the patients had fasted overnight. Laboratory data were determined using enzymatic methods. BP was determined as the mean of two measurements obtained in an office setting by the conventional cuff method using a mercury sphygmomanometer.

Data on weight, height and medication use were collected. Body mass index (BMI) was calculated as weight (kg)/height^2^ (m^2^). Medications included angiotensin II receptor blocker (ARB)/angiotensin converting enzyme inhibitor (ACE-I), calcium channel blocker (CCB), β-blocker, diuretics, statin, nitrate, nicorandil, oral hypoglycemic agent (OHA) and insulin. With regard to the use of antiplatelets, aspirin and ticlopidine or clopidogrel were generally administered. The patient characteristics, including the history of hypertension (HTN), dyslipidemia (DL), diabetes mellitus (DM) and smoking (current and past smokers), were obtained from medical records. Patients who had a current SBP/DBP ≥ 140/90 mm Hg or who were receiving antihypertensive therapy were considered to have HTN. Patients with LDL-C ≥ 140 mg/dL and/or TG ≥ 150 mg/dL or HDL-C < 40 mg/dL, or who were receiving lipid-lowering therapy were defined as DL. DM was defined using the Japanese Diabetes Society criteria.

### Assessments of BP parameters

We used the data from four clinic visits during the follow-up period and determined VVV in BP expressed as a standard deviation (SD) of average BP, average BP, and maximum and minimum BP.

### Statistical analysis

Statistical analysis was performed using SAS 9.3 (SAS Institute Inc.). Data are shown as the mean ± SD. Categorical variables were compared between the groups by a Chi-square analysis. The significance of differences between mean values was evaluated by an unpaired *t* test. A receiver-operating characteristic (ROC) curve analysis was used to determine the cut-off values of VVV in SBP and maximum SBP to distinguish between patients with and without MI in the ACS group at the highest possible sensitivity and specificity. A value of P < 0.05 was considered significant.

## Results

### Patient characteristics at baseline in all patients and the ACS and SAP groups

The patient characteristics at baseline in all patients and the ACS and SAP groups are shown in Table 1. The mean ages of all patients and the ACS and SAP groups were 68 ± 12 and 68 ± 11 and 70 ± 11 years, respectively. The ACS group had lower percentages of prior PCI and use of CCB and nitrate, and lower left ventricular ejection fraction (LVEF), and a higher percentage of use of β-blocker than the SAP group. The PCI procedure in the ACS group was significantly different from that in the SAP group. The ACS group had a higher percentage of bare-metal stent and a larger stent size than the SAP group. There were no significant differences in conventional coronary risk factors (BMI, DL, DM, smoking and eGFR) between the groups.

**Table 1:** Patient Characteristics at Baseline in All Patients and the ACS and SAP Groups

	All patients	ACS	SAP
No. of patients	176	50	126
Age, years	69 ± 11	68 ± 11	70 ± 11
Male, n (%)	143 (81)	43 (86)	100 (79)
BMI, kg/m^2^	24.0 ± 3.5	24.2 ± 3.5	24.0 ± 3.5
Smoking, %	106 (60)	30 (60)	76 (60)
Prior MI, n (%)	40 (23)	9 (18)	31 (25)
Prior PCI, n (%)	65 (37)	12 (24)*	53 (42)
Prior CABG, n (%)	13 (7)	5 (10)	8 (6)
SBP, mm Hg	130 ± 19	128 ± 22	131 ± 16
DBP, mm Hg	73 ± 14	76 ± 16	72 ± 13
DL, %	163 (93)	48 (96)	115 (91)
TG, mg/dL	133 ± 85	121 ± 74	138 ± 89
LDL-C, mg/dL	99 ± 31	103 ± 34	97 ± 30
HDL-C, mg/dL	46 ± 12	45 ± 11	47 ± 12
DM, %	69 (39)	17 (34)	52 (41)
HbA1c, %	6.3 ± 1.2	6.2 ± 1.3	6.3 ± 1.2
LVEF, %	60 ± 15	54 ± 16*	62 ± 14
eGFR	61 ± 21	65 ± 24	59 ± 20
Medications			
ARB/ACE-I	147 (84)	44 (88)	103 (82)
CCB	131 (74)	32 (64)*	99 (79)
β-blocker	66 (38)	27 (54)**	39 (31)
Diuretics	34 (19)	12 (24)	22 (17)
Statin	155 (88)	45 (90)	110 (87)
Nitrate	118 (67)	28 (56)*	90 (78)
Nicorandil	30 (17)	9 (18)	21 (17)
OHA	43 (24)	13 (26)	30 (24)
Insulin	7 (4)	2 (4)	5 (4)
Target vessels of PCI			
RCA/LAD/LCx/LMT/BG, n (%)	59/84/27/2/4 (34/48/15/1/2)	14/26/6/1/3 (28/58/12/6/2)	45/58/21/1/1 (36/46/17/1/1)
PCI procedure			
BMS/DES/POBA, n (%)	42/123/11 (24/70/6)	23/24/3 (46/48/6)*	19/99/8 (15/79/6)
Stent length of BMS or DES	22.0 ± 6.9	22.4 ± 5.8	21.9 ± 7.2
Stent size of BMS or DES	3.1 ± 0.4	3.2 ± 0.4*	3.0 ± 0.4

SAP: stable angina pectoris; ACS: acute coronary syndrome; BMI: body mass index; MI: myocardial infarction; CABG: coronary artery bypass graft; PCI: percutaneous coronary intervention; SBP: systolic blood pressure; DBP: diastolic blood pressure; DL: dyslipidemia; TG: triglyceride; LDL-C: low-density lipoprotein cholesterol; HDL-C: high-density lipoprotein cholesterol; DM: diabetes mellitus; HbA1c: hemoglobin A1c; LVEF: left ventricular ejection fraction; eGFR: estimated glomerular filtration rate; ARB: angiotensin II receptor blocker; ACE-I: angiotensin converting enzyme inhibitor; CCB: calcium channel blocker; OHA: oral hypoglycemic agent; RCA: right coronary artery; LAD: left anterior descending coronary artery; LCx: left circumflex coronary artery; LMT: left main trunk; BG: bypass graft; BMS: bare-metal stent; DES: drug-eluting stent; POBA: plain old balloon angioplasty. *P < 0.05, **P < 0.01 vs. SAP group.

### Clinical outcomes in all patients and the ACS and SAP groups

Table 2 shows the clinical outcomes in all patients and the ACS and SAP groups during the follow-up period. The numbers of patients who had MACEs (MI, TLR and death) in all patients and the ACS and SAP groups were 19 (six, 18 and one), seven (three, seven and zero) and 12 (three, 11 and one), respectively. There were no differences in MACEs (MI, TLR and death) between the groups.

**Table 2:** Clinical Outcomes in All Patients and the ACS and SAP Groups

	All patients	ACS	SAP
MACE, n (%)	19 (11)	7 (14)	12 (10)
MI, n (%)	6 (3)	3 (6)	3 (3)
TLR, n (%)	18 (10)	7 (14)	11 (9)
Death, n (%)	1 (0.6)	0 (0)	1 (0.8)

MACEs: major adverse cardiovascular events; MI: myocardial infarction; TLR: target lesion revascularization.

### Various BP parameters in all patients and the ACS and SAP groups

Various BP parameters in all patients and the ACS and SAP groups during the follow-up period are shown in Table 3. The average SBP and the maximum and minimum SBP in the ACS group were significantly lower than those in the SAP group.

**Table 3:** Various BP Parameters in All Patients and the ACS and SAP Groups

	All patients	ACS	SAP
VVV of SBP	10.2 ± 4.2	9.8 ± 4.9	10.3 ± 6.6
VVV of DBP	6.9 ± 4.4	6.7 ± 3.2	7.0 ± 4.8
Average of SBP, mm Hg	129 ± 13	125 ± 13*	130 ± 13
Average of DBP, mm Hg	73 ± 8	73 ± 9	73 ± 8
Maximum SBP, mm Hg	140 ± 17	136 ± 15*	141 ± 17
Maximum DBP, mm Hg	81 ± 11	80 ± 10	81 ± 12
Minimum SBP, mm Hg	117 ± 14	114 ± 15*	119 ± 13
Minimum DBP, mm Hg	70 ± 9	66 ± 9	66 ± 9

VVV: visit-to-visit variability; SBP: systolic blood pressure; DBP: diastolic blood pressure. *P < 0.05 vs. SAP.

### Various BP parameters in the ACS and SAP groups with and without MACE or MI

**Figure 1:**
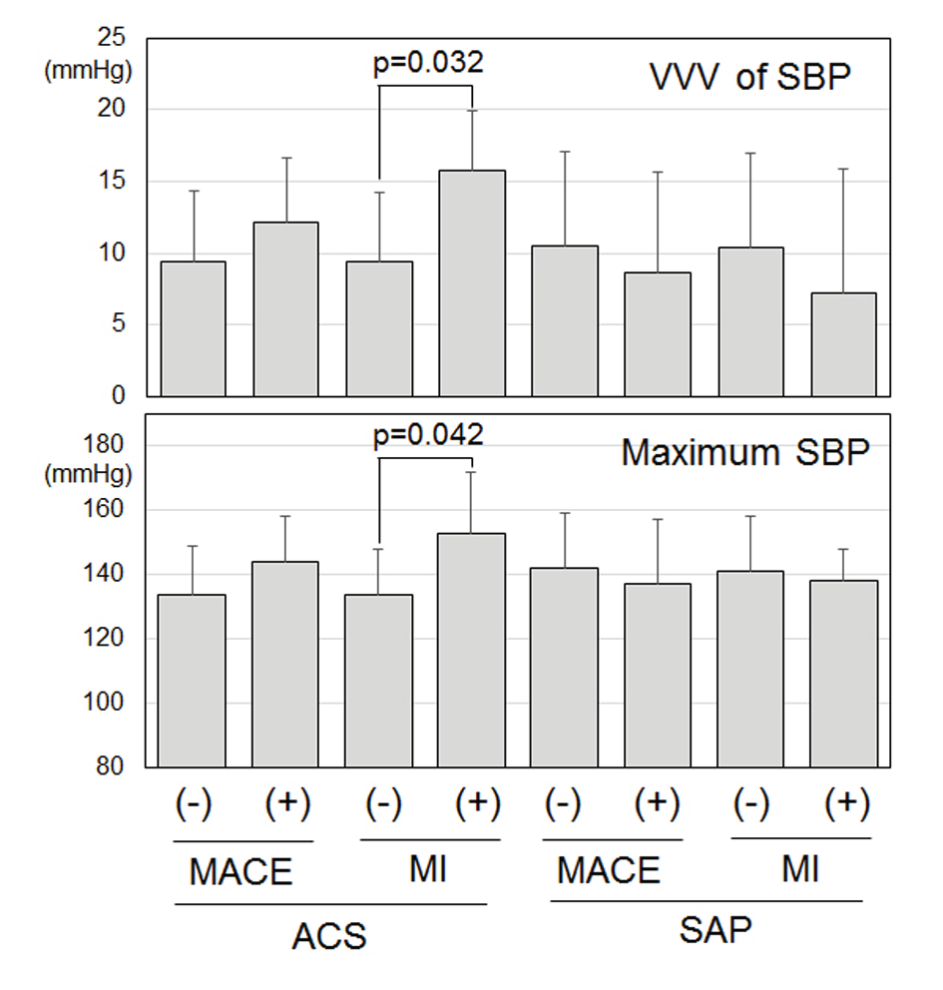
Visit-to-visit variability (VVV) in systolic blood pressure (SBP) and maximum SBP in patients with or without major adverse cardiovascular events (MACEs) and myocardial infarction (MI) in the acute coronary syndrome (ACS) and stable angina pectoris (SAP) groups.

Next, Figure 1 and Table 4 show various BP parameters in the ACS and SAP groups with or without MACE and TLR throughout the follow-up period. Interestingly, in the ACS group, the patients with MI showed significantly higher VVV in SBP and maximum SBP than the patients without MI (Fig. 1). There were no significant differences in other BP parameters between patients with and without MACE (Table 4).

**Table 4:** Various BP Parameters in All Patients, Patients With or Without MACE, TLR and MI in the ACS and SAP Groups

	ACS				SAP			
	MACE		MI		MACE		MI	
	(-)	(+)	(-)	(+)	(-)	(+)	(-)	(+)
VVV of DBP, mm Hg	6.6 ± 3.3	7.5 ± 3.0	6.7 ± 3.3	6.4 ± 3.0	7.2 ± 4.8	5.1 ± 3.8	7.1 ± 4.8	4.5 ± 3.1
Average of SBP, mm Hg	124 ± 13	130 ± 9	125 ± 13	135 ± 12	130 ± 13	129 ± 17	130 ± 13	130 ± 1
Average of DBP, mm Hg	73 ± 9	75 ± 8	73 ± 9	82 ± 6	73 ± 8	74 ± 7	73 ± 8	76 ± 3
Maximum DBP, mm Hg	79 ± 10	82 ± 11	79 ± 10	87 ± 12	82 ± 12	79 ± 10	81 ± 12	81 ± 2
Minimum SBP, mm Hg	114 ± 15	117 ± 10	114 ± 15	119 ± 14	119 ± 13	118 ± 13	119 ± 13	121 ± 12
Minimum DBP, mm Hg	66 ± 10	66 ± 8	65 ± 9	74 ± 4	66 ± 9	67 ± 6	66 ± 9	71 ± 6

VVV: visit-to-visit variability; SBP: systolic blood pressure; DBP: diastolic blood pressure; PP: pulse pressure.

### Cut-off values of VVV in SBP and maximum SBP to distinguish between patients with and without MI in the ACS group

An ROC curve analysis showed a higher area under the curve for a relative difference in VVV in SBP (0.83) and maximum SBP (0.83). The cut-off levels for VVV in SBP and maximum SBP that gave the greatest sensitivity and specificity for the presence of coronary thrombosis were 15.1 mm Hg (sensitivity 0.91, specificity 0.67) and 138 mm Hg (sensitivity 0.66, specificity 1.00), respectively.

## Discussion

In this study, we investigated the association between VVV in BP and MACE in hypertensive patients after successful PCI. The most important finding was that higher VVV in SBP and maximum SBP after successful PCI due to ACS were associated with the onset of MI. On the other hand, MACE was not associated with VVV in BP, average BP or maximum or minimum BP during the follow-up period.

We found that higher VVV in SBP and maximum SBP after successful PCI due to ACS may be predictors for the onset of MI. In addition, other BP parameters, including VVV in DBP, mean BP and minimum BP, were not associated with MI. Higher VVV enhances periodic pressure overload and shear stress on the CV system and contributes to the progression of atherosclerosis [16]. Disturbed shear stress could promote CVD pathogenesis by enhancing endothelial inflammatory and thrombotic responses [17]. In fact, two of the three patients who had MI in the ACS group in this study had coronary thrombosis. Thus, this could explain why VVV in SBP was associated with the onset of MI in the ACS group. We also found that maximum SBP after successful PCI may be a predictor of MI. Maximum SBP in addition to VVV in SBP was significantly associated with future risks of stroke and other CV events [3]. The maximum SBP in patients with MI in the ACS group was 153 ± 16 mm Hg and the cut-off level for the maximum SBP was 138 mm Hg, although the average SBP in the same patients was 135 ± 12 mm Hg and the levels of BP were under relatively good control because the target of BP control should be < 140/90 mm Hg according to the JSH2014 [10]. Since the maximum SBP was significantly associated with VVV in SBP in the ACS group (n = 50, r = 0.41, P = 0.004), the significance of maximum SBP may be similar to VVV in SBP. We need to notice transient SBP elevation and control BP < 140/90 mm Hg at every clinic visit.

VVV in SBP has been investigated more often than VVV in DBP. However, both were associated with adverse outcomes in a meta-analysis [4]. Although VVV in SBP was associated with MI in the ACS group, VVV in DBP was not associated with the clinical outcome. Patients who were revascularized in a coronary artery tolerated a lower DBP better than patients who were not revascularized [18]. Since we only enrolled patients who had undergone successful PCI, we may not have been able to observe the associations between CV events and VVV in DBP or mean DBP.

The number of visits, the time interval between visits, and the BP measurement protocols have varied widely across studies [4]. It has been reported that VVV in BP is influenced by the number of visits used to calculate it, the time interval between visits, the BP measurement device, and the number of BP measurements per visit [19, 20]. We enrolled patients who had undergone successful PCI and who had four clinic visits to measure BP until follow-up CAG at 6 - 9 months after PCI. Although VVV in BP was determined by the same number of visits for all patients (four times) and a similar time interval between visits (1 - 2 months) in this study, these factors may have affected the associations between VVV in BP and outcome. In addition, there is no consensus on how to calculate VVV in BP. Since the most common measures used to quantify VVV in BP were SD and coefficient of variation [4], we used SD. Further studies are needed to determine the VVV index, the number of visits and the time interval between visits that carry the great prognostic information.

MACE was not associated with BP parameters including VVV in BP in this study, although previous reports have indicated that VVV in BP was a strong predictor of CVD, stroke and mortality [1-4]. MACE occurred at a lower rate (11 %) and only one patient died throughout the study period. In addition, 18 of 19 patients (95%) with MACE had TLR. This could explain why VVV in BP was not associated with MACE.

There are several study limitations. First, the study included a relatively small number of patients. Second, the analysis was performed after various anti-hypertensive treatments, although there were no significant differences in the VVV in SBP and maximum SBP between patients with and without various medications (data not shown). Although aspirin and ticlopidine or clopidogrel were essentially administered with regard to the use of antiplatelets, some patients received warfarin. Third, we did not perform a logistic regression analysis due to the low percentages of MACE and MI. Prospective studies are needed to clarify these limitations.

In conclusion, higher VVV in SBP and maximum SBP in hypertensive patients with ACS were associated with the onset of MI after successful PCI.
